# Transdiscipline and research in health: science, society and decision making

**Published:** 2015-09-30

**Authors:** Fabián Méndez

**Affiliations:** Grupo de Investigación GESP. Escuela de Salud Pública. Universidad del Valle, Cali, Colombia

**Keywords:** Transdicipline, health, society, making decisions

## Abstract

Significant advances in science should be given to addressing the needs of society and the historical context of the territories. Although technological developments that began with modernity and the industrial revolution allowed human beings to control the resources of nature to put to your service without limits, it is clear that the crisis of the prevailing development models manifest themselves in many ways but with three common denominators: environmental degradation, social injustice and extreme poverty. Consequently, today should not be possible to think a breakthrough in the development of science without addressing global environmental problems and the deep social injustices that increase at all scales under the gaze, impassively in many occasions, of formal science.

## Abstract (es)

Los avances significativos en la ciencia deben darse de frente a las necesidades de la sociedad y al contexto histórico de los territorios. Aunque los desarrollos tecnológicos que empezaron con la modernidad y la revolución industrial permitieron al hombre controlar los recursos de la naturaleza para ponerlos a su servicio sin límites, es evidente que la crisis de los modelos de desarrollo predominantes se manifiestan de muchas formas en la sociedad actual y con tres denominadores comunes: deterioro del ambiente, injusticia social y pobreza extrema. En consecuencia, hoy no debería ser posible pensar un avance de relevancia en el desarrollo de la ciencia sin hacer frente a los problemas ambientales globales y a las profundas injusticias sociales que aumentan en todas las escalas bajo la mirada, en muchas ocasiones impasible, de las ciencias formales.

## Introduction

Major scientific progress must prioritize societal needs and the historical context of territories. Despite technological advances achieved by modernity and the industrial revolution, which have allowed man to control natural resources, placing them at his service without limits, it is clear that the crisis of predominant development models is present in many ways in current society. The crisis has three common denominators: environmental deterioration, social injustice and extreme poverty. Consequently, it is currently impossible to expect meaningful progress in scientific development without addressing global environmental issues and deep social injustices, which are increasing on all scales under the watchful eye, yet often indifferent, of the formal sciences.

In accordance with the previous statement, it is increasingly recognized the limitations of isolated disciplines for understanding and contributing to solve the issues of greatest concern. Complexity and uncertainty are currently recognized as two central characteristics of research, acknowledging that social and natural systems are complex and dynamic. Therefore, we need a science based on unpredictability, in which there is incomplete control and a diversity of legitimate perspectives 1.

There is also a current, increasing understanding that science without people, without other knowledge, and without considering the relationships with decision making will continue contributing to the accumulation of knowledge, but will be largely incapable of altering the life conditions of those in greatest need. In the history of humanity, we have never had so much evidence and technology available to positively influence peoples' quality of life and health. Yet paradoxically, at the same time, we had never, as today, been faced with fundamental issues of sustainability because of environmental deterioration and avoidable morbidity. Nonetheless, despite the need to develop an inclusive and comprehensive focus in science, to understand and participate in the health and wellness issues facing the majority of people, major scientific efforts continue to be uni-disciplinary.

This article reviews the historical background, definitions and several methodological aspects of transdisciplinary approaches and connects them to health research and its integration with new perspectives on development in science and decision making. Specifically, the review of published literature describes existing evidence on how to facilitate the development of transdisciplinary research to better understand health problems in a globalized world.

## Origins and developments

The production of knowledge has always occurred in a variety of institutions and places and not only in laboratories of academic spaces. However, with the growth of universities in the twelfth century, the division of knowledge was institutionalized, and the term "discipline" was born. The disciplines are distinguished by "having a particular objective of study and possessing a baggage of specialized knowledge regarding this objective, with theories and concepts that organize it, with specialized language, methods and institutional presence" 2. In the words of Barry et al., "disciplines discipline disciples" 3. Commitment to a discipline is a way of assuring that certain disciplinary methods and concepts are rigorously used, excluding the undisciplined and their methods, objectives and concepts. Along with this, and throughout the years, disciplines have increasingly moved towards specialization, isolating themselves from other fields of knowledge. Consequently, university organization today (i.e., by discipline) continues to be nearly the same as in the twelfth century.

The term "multidisciplinarity" appeared after the Second World War, in a particular historic moment with major changes in economic and political relations, in knowledge between nations 4 (in particular, relations between the world of rich nations and those declared poor) and as a need to establish links between theories of different disciplines 5. Specifically, multidisciplinary activities involve researchers from different disciplines who work independently, each one from the perspective of her own discipline, to address a common issue. Multidisciplinary approaches create an additional combination of knowledge but do not approach scientific integration. Consequently, the resulting product is the sum of all these efforts.

However, although the term "interdisciplinarity" first appeared in in the mid-1920s, it was commonly used in social sciences in the mid-twentieth century 6. It is understood that interdisciplinarity involves the transfer of methods from one scientific discipline to another, which to varying degrees makes reference to the interaction between disciplines but does not make an explicit call to transgress the limits between them.

The word "transdisciplinarity" was first mentioned in 1970 during the international workshop "Interdisciplinarity: Teaching and Research Problems in Universities". Erich Jantsch, an Austrian physicist, and Jean Piaget, the famous Swiss psychologist, adopted an interpretation referring to the theory of systems and a hierarchical model that positions multidisciplinarity beneath interdisciplinarity and the latter beneath transdisciplinarity 7. Jantsch and Piaget were in agreement in considering that multidiscipline only implied a juxtaposition of disciplinary knowledge, whereas interdiscipline suggested a coordinated and integrated approach between them. Specifically, Piaget wrote that transdiscipline "would not only cover interactions or reciprocities between specialized research projects, but would place these relationships within a total system without any firm boundaries between disciplines" 8. In other words, he suggested the need to transgress the disciplinary limits to achieve better science.

However, the idea and principles of transdisciplinarity, as an effort to think beyond academic disciplinary structures, is not new. Throughout history, in different moments, philosophers and scientists have demonstrated this need. Among them, it is noteworthy to highlight the appearance of quantum theory at the beginning of the twentieth century. Niels Bohr made a significant contribution to this theory, which arose at the start of a new era in physics and in the understanding of the world. Specifically, in the study of the phenomenon of light, Bohr asked about the wave-particle duality: in space, the movement of light is described as waves, but the effect of light as particles also constitutes a critical characteristic of light. Bohr addressed the issue from the point of view of complementarity in connection with contrasting observations obtained through mutually exclusive experiments. According to Bohr, the reason for this impossibility is that the study of light as waves or particles requires observation instruments, which are mutually exclusive, and consequently cause a basic limitation in the analysis of natural phenomena 9. This idea of complementarity is fundamental to transdisciplinarity because it acknowledges various levels of reality that, simultaneously, can explain a phenomenon from different viewpoints.

Beginning with philosophy, and inspired in the revolutions generated by quantum physics and the theory of relativity in the twentieth century, the Frenchman Gaston Bachelard described how traditional science evolved, simplifying reality in geometric structures, and made a call in favor of the development of abstract thought. He was one of the first to address the implications of the theories of Einstein and quantum theory in epistemology in the foundations of knowledge. He published his interpretations almost simultaneously regarding developments in physics. In the book, "The Formation of the Scientific Mind", Bachelard describes psychological barriers to developing a new form of science and suggests new ways to better understand the world 10.

In the natural sciences, Charles Darwin and his theory of evolution is another example of transdisciplinary research. Darwin articulated disciplines such as geology, biology, geography and genetics, with the goal of generating a theory of evolution. His work resulted in "The Origin of Species by Natural Selection," a book that was considered revolutionary in his time and served as a foundation for multiple fields of study, including evolutionary biology. In addition, from biology, Ludwig von Bertalanffy went beyond his own discipline and expanded his work to psychology, psychiatry, sociology, history and philosophy. Bertalanffy criticized analytical Cartesian thought and suggested that it could be replaced by a holistic approach or systems theory. He declared "the neccesity of investigating not only parts but also relations of organisation resulting from a dynamic interaction and manifesting themselves by the difference in behaviour of parts in isolation and in the whole organism" 11. 

Many varied contributions to the development of transdisciplines have occurred in recent decades, without arriving at a consensus. Edgar Morín thoroughly developed the concept of complexity as a central element of transdisciplinarity in his book, "The Method" 12, and in many of his writings on the evolution of disciplines. Other major contributions to the concept of transdisciplinarity include those of Basarab Nicolescu (International Center for Transdisciplinary Research - CIRET), Julie Thompson Klein (Wayne State University, Detroit, Michigan), Gertrude Hirsch Hadorn (University of Konstanz, Germany), Christian Pohl (Swiss Academy of Sciences), Daniel Stokols (School of Social Ecology, University of California), Helga Nowotny (European Research Council) and Atila Ertas (Texas Tech University), among others. 

In particular, Nicolescu affirms that transdisciplinarity refers to that which is *between* disciplines, *through* different disciplines and *beyond* all disciplines. According to Nicolescu, one of the imperatives of transdiscipline is the unity of knowledge, which is possible in the "discontinuous structure of transdisciplinary space" 13. In addition, Klein defines transdisciplinary approaches as "comprehensive frameworks that transcend the narrow scope of disciplinary world views through an overarching synthesis" 14. To achieve this, the transdisciplinary approaches accomplished by integrated teams seek a synthesis of research in their steps of conceptualization, design, analysis and interpretation. In this way, there is agreement that transdisciplinarity does not oppose disciplinary development but is against hyperspecialization.

Hirsch *et al*., however, emphasize cooperation from inside the scientific community and the necessary debate between research and society. These authors affirm that transdisciplinary research not only suggests a transgression of limits between scientific disciplines but also implies an analysis of the relationship between academics and society and includes a deliberation on facts, practices and values in sciences 15.

In other words, transdisciplinarity seeks an effective dialogue between traditional disciplines to strengthen academic communities with the goal of reinforcing the ability to generate, transmit, manage and apply knowledge. In that effective interdisciplinary dialogue, new disciplines can arise. Ecology, for example, is fundamentally based on a systemic vision of life. The study of ecosystems requires an understanding of physical, biological and social constituents, each one of these dependent on specialized disciplines, but which were finally joined together in a common language. In some way, in the words of Edgar Morin, ecology constituted "a new type of science" which, contrary to the dogma of hyperspecialization that has governed the development of scientific disciplines, is focused on a global knowledge that is competent in different domains 16.

Also, the development of schools of thought that bring together seemingly theoretically contradictory discipline is exemplified in the emergence of ecological economics. Specifically, ecological economics studies controversies that occur because of the monetization of the benefits of nature to propose alternatives that confront market logic and its negative effects on environmental conservation as a basis for human wellbeing 17. In this way, conflicts between disciplines that contradict one another in the current historical context seek theoretical and methodological solutions with academics that move from their formal fields of study towards new developments in science that adapt to societal needs.

## Transdisciplinarity in health

In the health field, the development of the model of the social determinants of health (SDH) implied an epistemological expansion of the object of study from different knowledge spheres and on different levels of analysis. Understanding health beyond biomedical risk factors requires contemplating scientific integration with other knowledge fields that analyze the life-health-disease-action process as a social phenomenon that should be studied in specific historical contexts. In this way, the SDH model implies an understanding of health as a complex phenomenon, and the development of effective actions requiring the integration of social, human and environmental sciences, as well as a major consideration of research implications in public policy.

In this way, health science contributions and limitations in decision making represent another issue that has been addressed by academics concerned by the weak relationship between health knowledge and public policy. Nevertheless, as Morello et al. suggested, the call to a "better" science, which often serves decision making, is used to reinforce sociopolitical and dominant economic systems because they slow down or paralyze decision making and prevent the necessary application of the precautionary principle in safeguarding peoples' health. In the debate to influence health politics, unfortunately, some "experts" assure that decision making remains "objective" and divorced from the socioeconomic and political context in which it occurs. The so-called "scientifization" of decision making, according to Morello, excludes the public from the debate and limits their capacity to participate in the production of knowledge 18.

In contrast, social movements those seek concessions, such as those because of problems of access to health services or social conflict struggles such as disputes over environmental issues that potentially affect health, have caused some investigators to propose an analysis of the role of science in its interaction with social and political arguments 18. The study of struggles for social concessions has characterized how social movements have influenced the duty of science and how this, in turn, provides foundations for new social movements. This constitutes an example of how academics cannot remain on the outside of the context of problems affecting communities. This type of research also incorporates scientific integration and the complementarity of quantitative and qualitative visions for a greater understanding of complex issues.

In particular, the public health field has always had a multidisciplinary nature because it is common to simultaneously require knowledge of biological, behavioral, health and social sciences to understand, explain and address a population's health problems. In accordance with the definition of health published several decades ago by the World Health Organization, it is acknowledged that current health issues cannot be addressed through medical science alone.

Additionally, public health has acknowledged the need to integrate other fields of knowledge. In particular, in Latin America there is a tradition of analyzing the social determinants of health from different schools of thought, such as social medicine, collective health, medical sociology and medical anthropology, which arose in the context of a social reality characterized by inequality. These inter/transdisciplinary academic movements have not only had to recognize, beyond biophysical risk factors, the relevance of different social aspects in the health-disease-action process, but they have also had to try to comprehend and support an organized social response.

Several figures and thinkers in Latin America have made fundamental contributions, on occasion without explicit mention or without global recognition, to the development of transdisciplinary thought. The so-called Latin American Social Medicine Movement, with the contributions of Asa Cristina Laurell, Edmundo Granda, Jaime Breihl, Jaime Samaja and Mario Testa, just to mention a few of the most well-known names, has worked on the integration of thought from philosophy, economics, sociology and anthropology to contribute to a contextual knowledge of health scenarios in our countries. In particular, another one of the great contributors has been Naomar de Almeida, who, in his book "Timid Science," proposed the deconstruction of epidemiology starting with the development of a theoretical model that integrates the complexity of the relationship between "way of life" and health. De Almeida proposed the integration of classic social epidemiology and its idea of risk, along with that of fundamental approaches not only in lifestyle but also in life conditions and social reproduction processes 19.

## Transdiscipline and methods: how to incorporate transdisciplinarity principles in research? 

"The world has problems, but universities have departments" 20. 

Various groups across the world are developing initiatives that seek to put transdisciplinary principles into practice. For example, there are fields of development in engineering and other disciplines that are attempting to establish methodological requirements for a transdisciplinary process to aid in decision making. The objective of these developments is focused on a cross-disciplinary approach to "joint problem-solving." Specifically, in the "Prevention Through Design" initiative, the need for engineer collaboration on social and natural sciences and humanities research has been suggested to "understand the impact on the environment and nearby communities of people to guide reiteration of their designs" 21.

In the public health field, the "Initiative in the Study and Implementation of Systems" (ISIS), led by the National Cancer Institute in the United States, was created with the goal of developing systemic thought to control tobacco consumption. Based on a transdisciplinary effort that connects tobacco-control actors and systems experts, ISIS combined a number of exploratory projects and case studies with a detailed examination of the potential of systemic thought on tobacco control. The final product was a series of guides for the future implementation of systemic thought and systems perspectives for public health and tobacco control 22.

However, given that there is no consensus on the conceptual framework defining transdisciplinarity, the expected process and transdisciplinary research methods also involve several approaches and goals. Different investigators in the transdisciplinary field argue in favor of different tasks and issues to be taken into account with the development of this type of research. Despite this, it is possible to identify various commonalities among them all. As Klein affirmed, it will be necessary to calibrate individual standards and carefully manage tension between different approaches, "balancing facts which require negotiation and compromise" 14. According to Klein, the evaluation of transdisciplinary research will require the definition of different and flexible principles including, among others, the coexistence of different goals, the establishment of new research quality indicators and the measurement of integration process levels among disciplines and communication levels with regional participants.

In accordance with these processes of transcending and integrating disciplinary paradigms, some have developed scales to measure collaborative processes and discipline integration. These "team-science" evaluation efforts seek to identify measure and understand collaboration processes and results on a large scale. In particular, Mâasse and colleagues developed and validated four scales that can be useful in transdisciplinary project implementation: three scales to evaluate collaborative processes (collaboration satisfaction, collaboration impact, confidence and respect) and one scale to evaluate transdisciplinary integration 23.

However, in addition to these useful evaluative approaches, one fundamental aspect that should be considered when conducting transdisciplinary research is the underlying epistemological theory. One significant qualitative challenge specifically relates to the development of complex thought. Specifically, Nicolescu explains that much confusion arises in the development of transdisciplinary research if the existence of three work areas is not acknowledged: the field of theoretical issues, the phenomenological field and the experimental field. According to Nicolescu, various groups around the world have attempted to develop transdisciplinary research, letting themselves be guided by one or another of these aspects.

Consequently, the groups working on transdisciplinary progress have developed two types of approaches, one that gives preference to joint problem-solving strategies and the other that seeks to solve epistemological problems. Although these approaches should be considered complementary, there is enormous tension between the two, perhaps because the epistemological approach suggests a change in the linear logic paradigm and breaks away from the premise of a single reality. Consequently, Max-Neef, taking sides, calls the first transdiscipline weak and the latter transdiscipline strong.

Additionally, another fundamental aspect in the incorporation of transdisciplinarity is the context in which this collaboration process is conceived of and developed. Collaboration, as described by Pohl, may be viewed as the result of joint work between two types of researchers: "detached specialists" and "engaged problem-solvers" 24. In a qualitative study on the practice of transdisciplinary groups, Pohl explains that if collaboration involves collaborative environments focused exclusively on the problem, this tends to take the form of division of labor. He concludes that in problem-oriented research, the pressure to produce useful results may be lessened so that true collaboration occurs. A desirable goal of fruitful collaboration should be the development of joint concepts among researchers. It is unsurprising, consequently, that this goal usually requires several years of collaboration to gain familiarity and develop respect for the 'culture' of other disciplines. Along with these factors described by Pohl, we would have to add similar factors with respect to other social participants, decision-makers and, in general, all those interested in, or potentially affected by, the research.

In this order of ideas and with the goal of concretizing methodological approaches that favor the development of transdisciplinarity, some have proposed the need to place greater emphasis on the way in which research problems are identified and structured. Specifically, Hirsch *et al*. 15, classify three types of knowledge: 1) systems knowledge, 2) objective knowledge and 3) transformation knowledge. The first of these three, systems knowledge, is most commonly generated by academic disciplines. It makes reference to the causes and future development of problems and holds as a major challenge the management of uncertainty in research results. Objective knowledge, as a complement, answers other questions related to people and ecosystems that concern us: What are the needs, interests and values of different participants that would be influenced by knowledge? The greatest challenge in this instance is the establishment of priorities and the definition of the common good in the midst of a diversity of positions. Finally, transformation knowledge refers to technical, legal, social, cultural and other types of strategies necessary to modify the current situation. The greatest challenge in this case, according to the authors, lies in identifying how these strategies can become more flexible amid existing practices and power relationships.

## Current situation and perspectives 

As a researcher in environmental epidemiology, I had the opportunity to develop an exploration on barriers and facilitators to transdisciplinary research in a group of environmental health researchers in Latin America 25. I specifically explored issues related to the formation, financing, execution, dissemination and use of project outcomes developed with a transdisciplinary approach. This exploration is summarized below.

In university training, unidisciplinary models are adhered to, hindering the development of more comprehensive perspectives. This is partly the result of university structures that favor hyper-specialization, making teamwork difficult. Consequently, the majority of researchers lack the conviction that transdisciplinary practices are better. Despite this, there are positive collaboration experiences that can serve as examples in training because they demonstrate how the research of complex issues is facilitated when work is performed in transdisciplinary teams. These experiences arose from the need to develop teaching strategies based on problem-solving. Those pedagogical approaches should also privilege the training of cross-disciplinary teams, curricular flexibility and interaction in training across research groups. One of the challenges of this training is the promotion of those who generate evidence corroborating the efficacy of transdisciplinary research.

Another issue that deserves analysis is related to the few opportunities of financing for projects with transdisciplinary approaches because most financing agencies favor more specific, unidisciplinary projects. In addition, it is likely that projects formulated with more integrative methodologies are not positively evaluated because external evaluators may not recognize the perspective and methods of this type of approach. Specifically, the design of these studies usually requires additional phases in which minimal agreements (e.g., - conceptual frameworks) are initially defined across different disciplines that are working together in an integrative way. This particularity, however, is precisely what allows for the incorporation of elements of other disciplines, enabling more integrative approaches for study aims.

For some, the execution of transdisciplinary projects should be performed in a way that articulates practical solutions to problems. However, it is no easy task to connect not only researchers of other disciplines but also other participants who share an interest in the research issue and who should be involved in research development. This, in part, is favored by a lack of understanding and respect for the culture, "style" and methods of other disciplines. In developing these types of projects, it is necessary to agree upon a definition of functions and responsibilities and to learn to manage inter-team conflicts with the goal of trust-building. In particular, we must build shared theoretical frameworks, recognizing the multidimensionality of health issues, to facilitate integrative development of research activities and de-stimulate the isolated work of team members.

It is not easy to publish the results of transdisciplinary research in publications with traditional approaches. This is one of the problems faced in the dissemination of those studies. Additionally, it is necessary to develop the means to communicate the results to different audiences, throughout the various project stages (not only at the end) and with various communication strategies that adequately integrate the results of different disciplines in publications. However, to utilize the results on a local or regional level, it will be necessary to work with different participants, from problem identification and study conception, to study interpretation and the definition of alternative solutions. Consequently, and clearly, in transdisciplinary projects, we must recognize other knowledge bases and visions in identifying alternative solutions.

## Conclusion

Transdisciplinarity not only refers to the work or joint use of different disciplines in integrated methodologies but it also involves the development of theories and, of course, a common language that integrates knowledge into a perspective of complementarity amid uncertainty. Transdisciplinarity also implies acknowledging, for our specific purpose, that health is a "contested territory" in which we must address the society-academia-decision-making relationship to have an effect on decision making.

The different definitions of transdisciplinarity and its corresponding methodological approaches result in tension among its users. Nonetheless, the application of these perspectives in health research is required when our intention is to understand the systemic roots of problems and to integrate diverse disciplines with the goal of solving these problems. Working together, however, is not enough to achieve innovative and effective approaches that help to intervene in complex health issues. Even more so, real collaboration is not the result of focusing exclusively on the acquisition of useful products but rather in constructing common, cross-disciplinary conceptual frameworks. The development of new, cross-disciplinary concepts would appear, then, to be a fundamental goal, which certainly can be achieved only after long-term work between experts in different knowledge areas.

The development of uni-disciplinary research projects increasingly determines the acquisition of hyper-specialized results that usually have a limited scope in understanding and participating in complex public health issues. In contrast, transdisciplinary research incorporates the involvement not only of academics from a variety of disciplines in defining and structuring the issue but also includes participants from outside the academic sector. The aim is for knowledge to have a greater involvement in defining action strategies and in formulating public policy. 
